# Patient Selection for Revascularization of Atherosclerotic Renal Artery Stenosis: Comparing the Importance of Stenosis Severity and Clinical Phenotype

**DOI:** 10.1016/j.xkme.2025.101213

**Published:** 2025-12-13

**Authors:** Darren Green, John G.F. Cleland, Hannah O’Keeffe, Rajkumar Chinnadurai, Edward Lake, Constantina Chrysochou, Philip A. Kalra

**Affiliations:** 1 Department of Renal Medicine, Salford Royal Hospital, Northern Care Alliance NHS Foundation Trust, Salford, UK; 2 Faculty of Biology, Medicine and Health, University of Manchester, Manchester, UK; 3 Department of Vascular Interventional Radiology, Manchester University NHS Foundation Trust, Manchester, UK; 4 School of Cardiovascular and Metabolic Health, University of Glasgow, Glasgow, UK

**Keywords:** Cardiovascular, chronic kidney disease, heart failure, renal artery stenosis, revascularization

## Abstract

**Rationale & Objective:**

Trials failed to show that angioplasty and stenting of atherosclerotic renovascular disease (ARVD) conferred benefit when used as first-line therapy. However, some patients might benefit from kidney revascularization depending on their clinical phenotype and severity of renal artery stenosis (RAS). We investigated this hypothesis further.

**Study Design:**

Data from the Angioplasty and Stenting for Renal Artery Lesions (ASTRAL) randomized trial and Salford ARVD observational study were included in a single analysis.

**Setting & Participants:**

Patients were grouped based on RAS severity (≥70%) and whether unilateral or bilateral, with the bilateral group including RAS in a single functioning kidney. High-risk clinical phenotypes included advanced chronic kidney disease (CKD) (estimated glomerular filtration rate < 30 mL/min/1.73 m^2^), rapid CKD progression (creatinine increase >100 μmol/l or >20% per year), refractory systolic hypertension (≥150 mm Hg on ≥3 agents), and heart failure (chronic or decompensated).

**Exposures:**

Medical therapy alone versus medial therapy and kidney revascularization.

**Outcome:**

Composite of end stage CKD, cardiovascular events, or all-cause mortality.

**Analytical Approach:**

Cox proportional hazard model adjusted for age, self-reported gender, and estimated glomerular filtration rate. Analysis of all patients and selected subgroups.

**Results:**

In total, 1,644 patients (806 ASTRAL and 838 Salford). Median (IQR) age 72 (66-77) years. For bilateral severe RAS (≥70%) the HR for the composite outcome for revascularization compared with medical therapy was 0.70 (0.50-0.99), *P* = 0.048. The clinical phenotype where benefit appeared to be greatest in the presence of bilateral severe disease was people with rapidly progressive kidney disease with a HR of 0.39 (0.22-0.71]). In the absence of bilateral severe RAS, there was no benefit to revascularization for any clinical phenotype.

**Limitations:**

The analyses included observational data.

**Conclusions:**

The presence of bilateral severe RAS may be the best predictor of benefit for kidney revascularization.

## Plain-language Summary

Previous clinical trials have indicated that undertaking procedures to restore blood flow to narrowed arteries in the kidneys should not be used routinely in patients with this condition (renal artery stenosis). This study looked at whether the procedure would benefit some patients, namely those with high-risk features. It used data from more than 1,000 patients, some of whom had the procedure and some who did not. It found that people with severe narrowing in both kidneys, particularly those who also had heart failure, had better long-term outcomes if they had the procedure. This helps us better understand when the procedure should be used.

Atherosclerotic renovascular disease (ARVD) is a common cause of chronic kidney disease (CKD), present in up to 13% of patients in hospital nephrology clinics and 13% of patients undergoing angiography for suspected or known coronary disease. ARVD is associated with poor outcomes, primarily because of high rates of cardiovascular events as atheroma is often widespread.^1^, ^2^, ^3^

Renal artery stenosis can contribute directly to cardiovascular disease onset and progression when hemodynamically significant. This is associated with neurohormonal overactivation via increased release of renin with downstream increases in production of angiotensin II and aldosterone. The result is greater risk of hypertension, sodium and water retention, adverse cardiac remodeling and chronic inflammation.^4^^,^^5^ This pathophysiology is consistent with findings in observational studies demonstrating profound hypertension, heart failure, left ventricular hypertrophy, and worsening of kidney function in ARVD compared with other causes of CKD.^3^^,^^6^ It is these factors that have driven recommendations on when to consider revascularization in atherosclerotic renal artery stenosis. The Kidney Diseases: Improving Global Outcomes (KDIGO) guidelines consider revascularization appropriate if typical high risk cardiovascular phenotypes are present such as refractory hypertension, rapidly declining kidney function, or acute pulmonary edema including decompensation of heart failure. In addition, these guidelines recommend consideration of intervention when stenoses of greater severity are present.^7^ The hemodynamic effects of renal artery stenosis typically become clinically significant when stenosis approaches 70%-80%, hence explaining why those with severe disease are most likely to derive benefit.^7^^,^^8^ In patients with ARVD and heart failure, increased angiotensin leads to vasoconstriction and increased proinflammatory cytokine production, which drives left ventricular hypertrophy and cardiac remodeling.^5^^,^^9^ Those with ARVD and heart failure, in particular who have had flash pulmonary edema, are considered for revascularization with the rationale thought to be due to a resultant improvement in the neurohormonal dysregulation.

Clinical trial findings to support these recommendations are lacking. The Angioplasty and Stenting for Renal Artery Lesions (ASTRAL) trial investigated revascularization of renal artery stenosis versus medical therapy for patients with renal artery stenosis ≥50%. The primary outcome measure was change in kidney function over time, and no difference was found between trial arms.^10^ A criticism of the trial was that it included very few high-risk patients (eg, heart failure prevalence 5% in ASTRAL compared with 15%-20% in renovascular clinical practice) and that the primary outcome measure did not accommodate the broader potential cardiovascular benefit of revascularization. Despite tighter inclusion criteria and core radiological adjudication of stenosis, the Cardiovascular Outcomes in Renal Atherosclerotic Lesions (CORAL) trial similarly found no significant difference between revascularization and medical therapy. The primary end point in CORAL was a composite of kidney and cardiovascular outcomes.^11^ Intervention is not benign, and risks of revascularization in these studies were predominantly procedure related (9% of revascularized ASTRAL participants and 5% of revascularized CORAL participants), including arterial dissection, perforation or aneurysm, and kidney or cholesterol emboli.^10^^,^^11^

The aim of these analyses was to explore the hypothesis that the severity of renovascular disease at which kidney revascularization is indicated is dependent on the phenotypic risk profile of the patient. Specifically, we considered whether high-grade disease should be considered for revascularization regardless of phenotype given the longer-term risk associated with hemodynamically significant disease and whether high clinical risk phenotypes may benefit from revascularization in the presence of less severe disease.

## Methods

Two sources of data were used. First the trial dataset from ASTRAL, a randomized control trial (RCT) of revascularization and medical therapy versus medical therapy alone in 806 people with ARVD ≥50% in at least 1 kidney, which recruited 2001-2008 with patients providing written informed consent to participate (ISRCTN59586944, ethics permission from West Midlands United Kingdom Multicenter Research Ethics Committee). ASTRAL reported in 2009 with a long-term outcome follow-up study published in 2024.^10^ The second data source was the Salford ARVD observational cohort study (ethics permission from Salford & Trafford Local Research Ethics Committee UK, reference 07/Q1404/44). Here, all patients with a radiological diagnosis of ARVD who were referred to the Salford tertiary center were retrospectively included in a pseudonymized cohort study (1990-2021) that did not require individual participant consent. There were 876 participants at the time of this analysis.^12^^,^^13^ This research was undertaken in accordance with the Declaration of Helsinki.

In both studies, diagnoses of ARVD were based on local imaging, overwhelmingly magnetic resonance or computerized tomographic angiography. Estimation of severity of stenosis was based on percentage narrowing of luminal diameter from diagnostic imaging. Of note, 22% of patients in the ASTRAL trial revascularization arm did not undergo the procedure, most often because disease was found to be nonsignificant during confirmatory on-table direct angiography.

For the present analysis, all participants in both studies were included. Phenotype data collected were those available from both cohorts: age, self-reported gender, cardiovascular comorbid conditions (heart failure, diabetes, coronary artery disease, smoking status), baseline measured parameters (estimated glomerular filtration rate (eGFR), systolic blood pressure, number of antihypertensive drugs), selected baseline drugs, and degree of stenosis in each dominant renal artery expressed as percentage of luminal diameter. Measures of proteinuria were not included as they were present in fewer than half of patients. Definitions of variables were the same in both datasets allowing integration.

Subgroups for analysis based on high risk phenotypes were created according to the following criteria: advanced CKD (eGFR <30 mL/min/1.73 m^2^ at baseline), refractory hypertension (systolic blood pressure [SBP] ≥150 mm Hg on 3 or more antihypertensive drugs), heart failure (clinical or imaging diagnosis of heart failure made by the responsible clinician or past hospitalization for heart failure, acute pulmonary edema, or flash pulmonary edema), rapid progressor of kidney dysfunction (increase in the serum creatinine level of more than 20% or of more than 100 μmol/L in the 12 months before recruitment), cardiometabolic risk (known diagnoses of both coronary disease and diabetes mellitus). Patients were classified as non-high risk if none of these features were present.^14^, ^15^, ^16^, ^17^

Subgroups for analysis were also formed based on both stenosis severity and whether unilateral or bilateral. Severe stenosis was defined as stenosis ≥70% in a dominant artery, and “moderate or severe” stenosis as ≥50%. Patients with stenosis supplying a single functioning kidney were included in the “bilateral” group. This is because in almost all renovascular disease cases, single functioning kidneys are due to renal artery occlusion in the contralateral organ leading to atrophy and loss of filtration capacity, rather than congenital or surgical single kidneys. Groups were mutually exclusive, with allocation being made based on most severe stenosis if severity differed between left and right.

**Figure 1:**
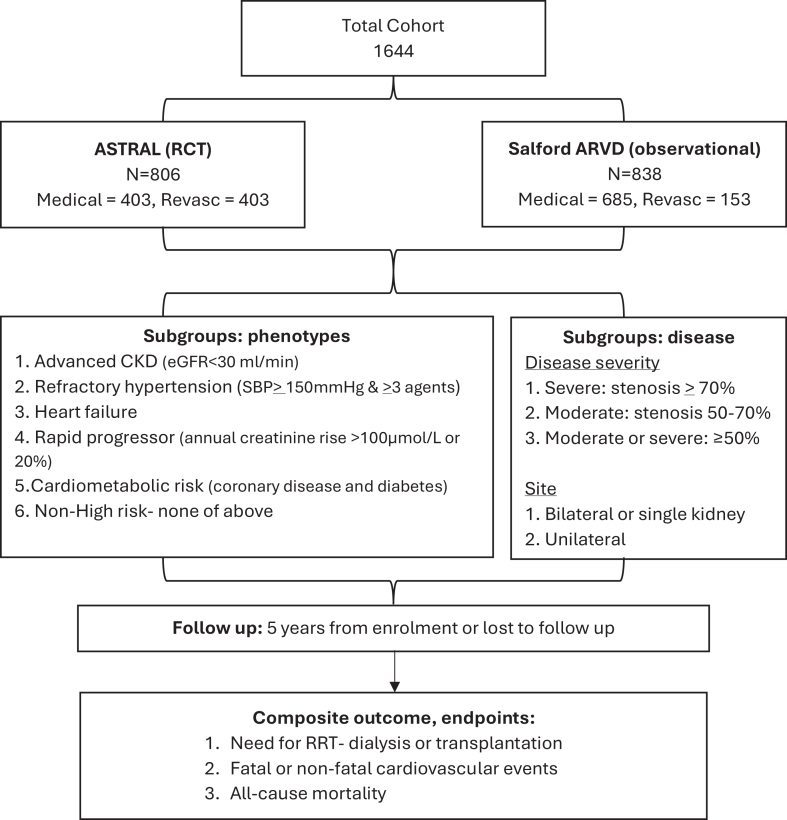
Flowchart of patient selection to the study.

Follow-up was for up to 5 years from enrolment into either study or until mortality or lost to follow-up, whichever came first. A composite cardiorenal outcome was used. End points included were end stage kidney disease, defined as the need for kidney replacement therapy including dialysis or transplantation (or eGFR <15 mL/min/1.73 m^2^ if for conservative care only); fatal or nonfatal cardiovascular event including de novo diagnoses of, or intervention for and hospitalization for existing coronary artery disease, arrhythmia, heart failure, stroke, and peripheral artery disease; and all-cause mortality. A flowchart illustrating patient selection is shown in Figure 1.

Time to event analyses were undertaken comparing patients undergoing revascularization alongside standard of care medical therapy versus those on medical therapy alone. A Cox proportional hazard model was used to determine hazard ratios (HRs) for outcome in the revascularization group versus those on medical therapy (reference group). These were adjusted for comorbid factors found to be statistically significantly (*P* < 0.05) associated with the composite outcome on univariate analysis within the cohort being analyzed, described below. Results of these univariate analyses detailing factors entered into multivariable models are found in Table S1.

Analyses were performed on subgroups according to stenosis severity and clinical phenotype and were undertaken from the ASTRAL randomized trial group alone and then in a combined ASTRAL and Salford cohort dataset. ASTRAL patients were included in the revascularization group based on intention to treat within the trial; therefore, this included trial patients in whom no significant disease was found during direct angiography.

Undertaking 2 sets of analyses in this way was intended to reflect that the analysis of only ASTRAL trial results would be from a randomized clinical trial, albeit post hoc. The inclusion of Salford study patients in the second set of analyses was to overcome the fact that the ASTRAL trial had limited representation of high-risk clinical phenotypes and no characterization of baseline existing heart failure diagnoses. Therefore, analysis of ASTRAL data was undertaken in subgroups of people with or without any high-risk clinical phenotype, creating clinically high risk and low risk groups. Whereas analysis of each specific high-risk phenotype was possible in the ASTRAL and Salford combined cohort.

Tests for interaction between clinical phenotype (presence or absence of any high-risk phenotype) and intervention were performed using likelihood ratio tests in analyses of ASTRAL data. Tests for heterogeneity of outcomes between each of the specific high-risk phenotypes were performed in the combined ASTRAL and Salford population by deriving a Q statistic from the hazard ratio and confidence intervals of each phenotype subgroup analysis.

Analyses were undertaken using SPSS, version 23 (IBM). Cases with missing values were excluded from analyses, not replaced with imputed estimations. No funding or other support was received for this work.

## Results

There were 1,644 patients in the cohort for analysis, including 806 from ASTRAL and 838 from the Salford cohort study. In total, 38 patients from the Salford cohort were excluded because of incomplete outcome data. The overall median age was 72 years (range 40-92 years). In total, 795 (48%) had existing coronary disease at enrolment or recruitment, and 501 (30%) had diabetes. More than 70% of the cohort were current or exsmokers. In total, 199 (24%) of the Salford cohort had heart failure or an episode of acute pulmonary edema not defined as heart failure. Similar heart failure data were not available in the ASTRAL trial dataset.

The most common high-risk phenotype was advanced CKD, with 714 (43%) of people having stage 4 CKD. In total, 532 (33%) of patients had refractory hypertension, and 279 (17%) had both diabetes and coronary disease. The overall median baseline kidney function (eGFR) was 32 mL/min/1.73 m^2^ (range 3-96 mL/min/1.73 m^2^), systolic blood pressure 150 mm Hg (range 80-270 mm Hg), and number of antihypertensive agents 3 (range 0-7).

Features were broadly similar between the ASTRAL and Salford populations (Table 1), except for a higher proportion of rapid progressors in the Salford observational dataset (39% versus 12% in ASTRAL). Salford patients also had a higher annual mortality (13% per year versus 9% per year in ASTRAL). Median follow-up was 38 months (IQR 13-60 months) for the ASTRAL cohort and 39 months (IQR 13-60 months) for the Salford cohort. Univariate analysis of each baseline variable with the composite outcome, for ASTRAL data alone, Salford data alone, and for the combined cohort are found in Table S1. These indicate which variables were included in the final multivariable models, results for which are described below.

**Table 1:** Baseline characteristics of patients included in the analyses. Data are divided according to source and whether people were managed with medical therapy alone or with medical therapy plus kidney revascularization.

Feature	ASTRAL		Salford	
	Medical	Revasc	Medical	Revasc
N (% of total cohort)	403 (24.5)	403 (24.5)	685 (42)	153 (9)
Age (years)^a^	71 (67-77)	72 (67-77)	72 (65-77)	69 (63-74)
Male, n (%)	253 (63)	254 (63)	363 (53)	77 (50)
Comorbid conditions				
Coronary disease, n (%)	189/391 (48)	192/387 (50)	298/682 (44)	81/148 (55)
Diabetes mellitus, n (%)	115/391 (29)	121/387 (31)	216/683 (32)	45/148 (30)
Heart failure, n (%)	na	na	141/677 (21)	52/146 (36)
Rapid progressor, n (%)	48 (12)	49 (12)	115/312 (37)	67 (44)
eGFR <30 mL/min, n (%)	179/391 (44)	165/387 (40)	275/617 (45)	69 (45)
Refractory hypertension, n (%)	149/391 (36)	153/387 (37)	172/647 (27)	50/145 (34)
Current or exsmoker (%)	301/391 (77)	276/387 (71)	278/385 (72)	54/76 (71)
Medicines				
ACEi or ARB, n (%)	146/388 (38)	174/384 (45)	334/682 (49)	74/150 (49)
Beta-blocker, n (%)	200/388 (52)	172/384 (45)	238/682 (35)	61/150 (41)
Antiplatelet, n (%)	298/383 (78)	289/381 (76)	360/682 (53)	89/150 (59)
Lipid-lowering, n (%)	312/389 (80)	304/381 (80)	360/682 (53)	86/150 (57)
Disease severity				
Systolic BP (mm Hg)^a^	150 (133-170)	146 (131-162)	150 (133-172)	160 (140-186)
eGFR (mL/min/1.73 m^2^)^a^	33 (24-42)	33 (23-44)	32 (21-45)	32 (21-43)
Bilateral severe RAS, n (%)	106 (26)	108 (27)	41 (6)	43 (28)
Unilateral severe RAS, n (%)	212 (52)	212 (52)	260 (38)	72 (47)
Outcomes				
KRT, n (%)	58 (14)	53 (13)	82 (12)	20 (13)
Death, n (%)	139 (34)	132 (32)	329 (48)	57 (37)
CVE, n (%)	145 (35)	136 (33)	185 (27)	44 (29)
Any event, n (%)	231 (57)	221 (54)	404 (59)	92 (60)

Abbreviations: ACEi, angiotensin-converting enzyme inhibitor; ARB, angiotensin receptor blocker; BP, blood pressure; CVE, cardiovascular event; eGFR, estimated glomerular filtration rate; KRT, kidney replacement therapy; na, not available; RAS, renal artery stenosis.

In ASTRAL the features of patients randomized to kidney revascularization or not were similar. Within the Salford cohort, revascularized patients were younger than those treated medically, had higher systolic blood pressure, and were more likely to have severe RAS. Only 18% of the Salford observational cohort patients underwent revascularization.

### ASTRAL Trial Data Alone

In the time to event outcome analyses of patients from ASTRAL, there were 214 patients with bilateral severe disease ≥70% or severe disease in a single functioning kidney. The adjusted likelihood of a composite outcome in patients undergoing revascularization compared with those with medical therapy (regardless of risk phenotype) was 0.7 (0.5-0.9), *P* = 0.048. The model was adjusted for age, coronary disease, smoking, angiotensin-converting enzyme inhibitor (ACEi) or angiotensin receptor blocker (ARB) use, beta-blocker use, antiplatelet use, and eGFR (Table S1). The annualized rate for cardiovascular events was 16.1% in medically managed patients and 12.5% in those in the revascularization group. In the high-risk phenotype subgroup, the figures were 19.6% and 13.8%, respectively. The annual rate of death in the overall medical group was 10.4% and 8.2% in the revascularization group. In the high-risk phenotype patients, the respective figures were 10.7% and 8.7% (Table 2 and Table S2).

**Table 2:** Annualized event rates (percent) and hazard ratios for the composite outcome of cardiovascular and kidney events or all-cause mortality in ASTRAL trial participants undergoing renal revascularization alongside standard of care, versus those only managed with medical standard of care. Findings are adjusted for baseline age, gender, and kidney function, and displayed according to subgroups of clinical phenotypes and stenosis severity.

Population	N		Any event (n, %)		Annualized event rate (any event)^a^		HR (95% CI)	*P*	*P* (interaction)
	Medical	Revasc	Medical	Revasc	Medical	Revasc			
ASTRAL bilateral severe stenosis ≥ 70%									
All patients	106	108	70 (66)	62 (57)	25.9	19.7	0.70 (0.50, 0.99)	0.048	0.801
High risk phenotypes	83	85	59 (71)	52 (61)	29.8	22.1	0.70 (0.48, 1.03)	0.07	-
Non-high risk	23	23	11 (47)	11 (47)	15.2	13.6	0.50 (0.20, 1.28)	0.15	-
ASTRAL bilateral moderate or severe stenosis (≥50%)									
All patients	202	188	129 (63)	112 (59)	24.6	21.0	0.85 (0.66, 1.10)	0.218	0.116
High risk phenotypes	161	145	108 (67)	96 (66)	27.1	25.3	0.91 (0.69, 1.21)	0.522	-
Non-high risk	41	43	21 (51)	16 (37)	16.9	10.4	0.58 (0.30, 1.15)	0.121	-
ASTRAL unilateral severe stenosis ≥70% with any disease <70% in contralateral kidney									
All patients	212	212	118 (55)	117 (55)	18.2	19.5	1.06 (0.82, 1.37)	0.663	0.168
High risk phenotypes	160	138	100 (62)	85 (61)	21.1	24.0	1.09 (0.81, 1.46)	0.568	-
Non-high risk	52	74	18 (34)	32 (43)	10.3	13.1	1.27 (0.70, 2.03)	0.44	-

Abbreviations: HR, hazard ratio.

a % per year.

Within the bilateral severe stenosis group, the hazard ratio for the composite outcome in patients with a high-risk clinical phenotype undergoing revascularization was 0.70 (0.48-1.03), *P* = 0.07 (n = 168). In the remainder (non-high risk) there was no statistical difference (0.50 (0.20-1.28), *P* = 0.150). The likelihood ratio test to determine interaction between phenotype and intervention was nonsignificant (Table 2).

Results for the effect of intervention were not statistically significant in the total group of patients with moderate or severe disease overall (0.85 (0.66-1.10), *P* = 0.218, n = 390), nor for moderate or severe disease with high-risk phenotypes. In ASTRAL patients with unilateral severe disease, there was no significant difference in outcome between people revascularized or medically managed irrespective of the presence or absence of a high-risk clinical phenotype. Analyses from the ASTRAL trial for people with each high risk clinical phenotype were limited by small sample sizes. The full findings for the ASTRAL only analyses are shown in Table 2 and Table S2.

### ASTRAL and Salford Combined Analysis

**Figure 2:**
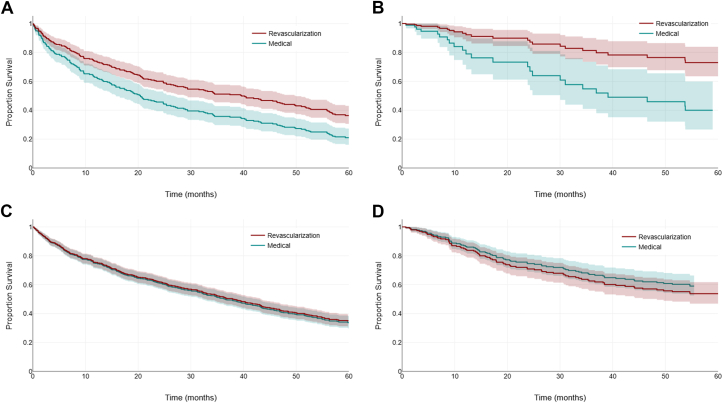
Kaplan–Meier time to event curves comparing composite outcomes in revascularization versus medical therapy alone for subgroups of renovascular disease based on disease severity and clinical phenotype, ASTRAL and Salford combined. (A) Bilateral severe disease with a high-risk clinical phenotype. (B) Bilateral severe disease without a high-risk clinical phenotype. (C) Unilateral severe disease with a high-risk clinical phenotype. (D) Unilateral severe disease without a high risk clinical phenotype.

In the combined analysis people with bilateral severe disease, regardless of risk phenotype, who underwent revascularization (n = 152) had a HR for the composite outcome of 0.59 (0.49-0.80), *P* = 0.001 compared with medically managed patients. This was adjusted for age, coronary disease, ACEi or ARB use, beta-blocker use, lipid-lowering therapy, and eGFR (Table S1). In people with bilateral severe disease and a high-risk phenotype, the HR was 0.60 (0.43-0.82), *P* = 0.002. The clinical phenotype where benefit appeared to be greatest in the presence of bilateral severe disease was the subgroup with rapidly progressive kidney dysfunction, with a HR of 0.39 (0.22-0.71). Tests for heterogeneity between outcomes in different phenotype subgroups were all nonsignificant, indicating that the findings in each phenotype could be considered similar. Kaplan–Meier curves for each of the high risk clinical phenotypes are found in Figure 2, comparing both medical therapy versus revascularization and also dividing results by cohort (ASTRAL and Salford). In the subgroup of people with bilateral severe disease but without a high-risk phenotype (n = 63), the HR was 0.41 (0.17-0.96), *P* = 0.04.

**Figure 3:**
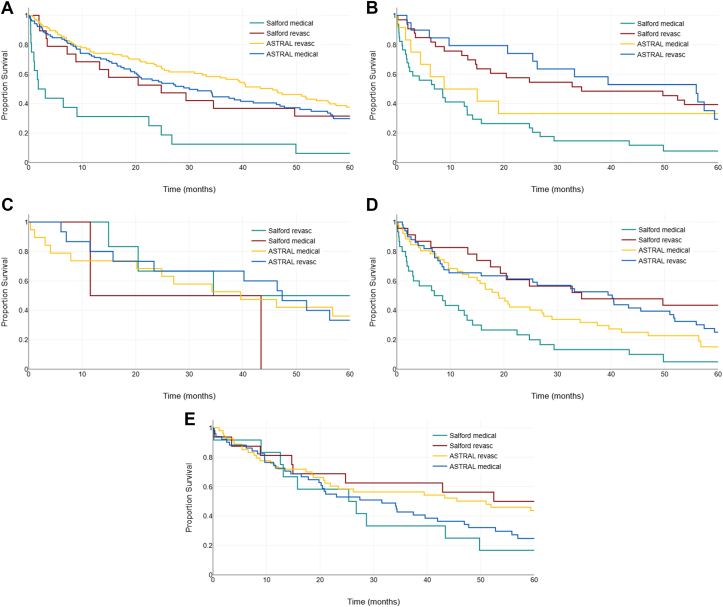
Kaplan–Meir curves for all high-risk clinical phenotypes, separated between revascularization and medical therapy, and comparing these interventions between the ASTRAL and Salford cohorts. (A) Heart failure. (B) Rapid progressor. (C) Coronary artery disease and diabetes mellitus. (D) Advanced chronic kidney disease. (E) Refractory hypertension.

**Figure 4:**
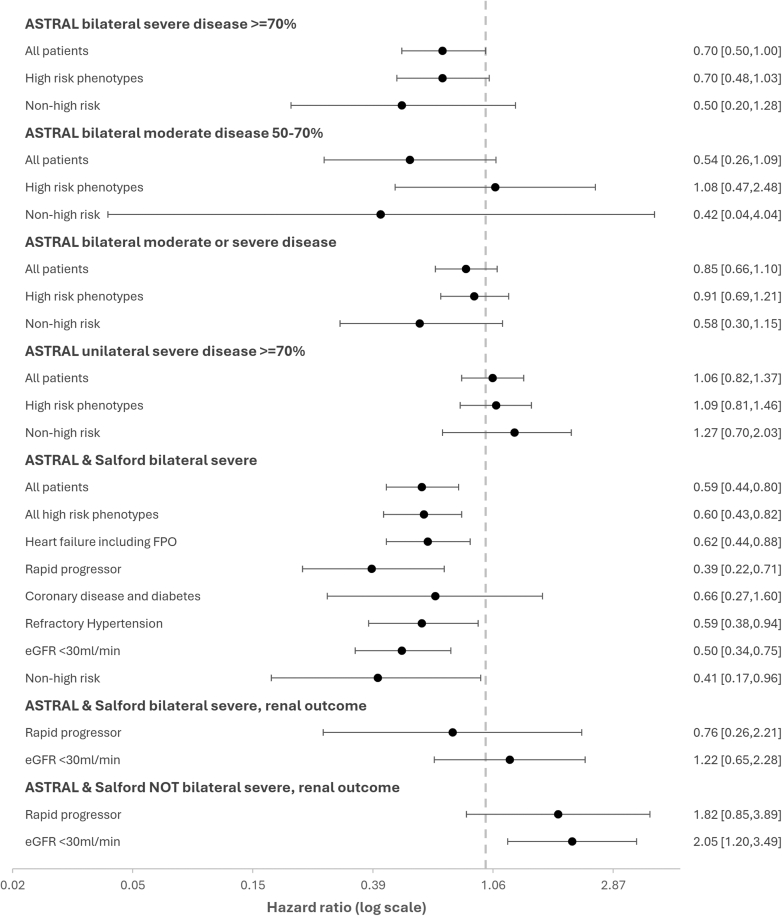
Forest plots comparing kidney revascularization versus medical therapy in all patient groups. Abbreviations: eGFR, estimated glomerular filtration rate; FPE, flash pulmonary edema.

In the combined analysis of people where severe disease was not present in either kidney (unilateral or bilateral disease of <70% stenosis), there was no benefit to revascularization in any phenotype group. Full findings from the combined group analyses are found in Table 3 and Table S3. Further analyses of only the ASTRAL patients and only the Salford patients from within this cohort are found in Table S4. All tests for interaction between phenotype and intervention were nonsignificant. Accompanying Kaplan–Meier survival curves are seen in Figure 3, displaying cumulative event rates for people with bilateral severe disease with (Fig 3A) and without (Fig 3B) a high-risk phenotype. Figure 3C and D show event rates for unilateral severe disease patients with and without high-risk clinical phenotypes, respectively. Figure 4 includes forest plots to compare HR, and confidence bands for revascularization versus standard of care in selected patient groups are found in Tables 2 and 3.

**Table 3:** Annualized event rates (percent) and hazard ratios for the composite outcome of cardiovascular and Kidney Events or all-cause mortality in a combined group from the ASTRAL trial and Salford observational cohort undergoing renal revascularization alongside standard of care, versus those only managed with medical standard of care. Findings are adjusted for baseline age, gender, kidney function, and other variables as per Table S1, and displayed according to subgroups of clinical phenotypes and stenosis severity.

Population	N		Any event (n, %)		Annualized event rate (any event)^a^		HR (95% CI)	*P*	*P* (interaction)
	Medical	Revasc	Medical	Revasc	Medical	Revasc			
ASTRAL and Salford bilateral severe stenosis ≥ 70%									
All patients	151	152	105 (69)	84 (55)	32.1	19.5	0.59 (0.44, 0.80)	0.001	0.805
All high-risk phenotypes	119	121	86 (72)	71 (58)	34.8	21.5	0.60 (0.43, 0.82)	0.002	-
Heart failure including FPE	16	20	15 (93)	13 (65)	85.8	27.2	0.62 (0.44, 0.88)	0.008	-
Rapid progressor	17	27	11 (64)	17 (62)	27.6	19.8	0.39 (0.22, 0.71)	0.002	-
Coronary disease and diabetes	21	23	14 (66)	13 (56)	23.0	18.2	0.66 (0.27, 1.60)	0.361	-
Refractory Hypertension	60	70	44 (73)	37 (52)	28.7	17.9	0.59 (0.38, 0.94)	0.025	-
eGFR <30ml/min	70	77	62 (88)	45 (58)	47.7	23.8	0.50 (0.34, 0.75)	0.001	-
Non-high risk	32	31	19 (59)	13 (41)	23.7	12.8	0.41 (0.17, 0.96)	0.04	-
ASTRAL and Salford bilateral moderate or severe stenosis ≥ 50%									
All patients	381	277	232 (60)	157 (56)	25.4	20.5	0.82 (0.67, 1.01)	0.061	0.111
All high-risk phenotypes	298	212	195 (65)	134 (63)	27.9	24.4	0.86 (0.69, 1.08)	0.187	-
Heart failure including FPE	39	32	35 (89)	20 (62)	75.0	29.7	0.80 (0.62, 1.02)	0.076	-
Rapid progressor	54	41	34 (62)	27 (65)	22.0	24.4	0.77 (0.53, 1.12)	0.176	-
Coronary disease and diabetes	65	45	43 (66)	28 (62)	25.3	22.1	0.83 (0.50, 1.36)	0.451	-
Refractory Hypertension	145	113	89 (61)	64 (56)	23.5	19.6	0.86 (0.62, 1.19)	0.357	-
eGFR <30ml/min	182	123	128 (70)	84 (68)	34.0	28.4	0.77 (0.58, 1.02)	0.073	-
Non-high risk	83	62	40 (48)	23 (37)	17.6	10.6	0.73 (0.43, 1.25)	0.251	-
ASTRAL and Salford unilateral severe stenosis ≥70% with any disease <70% in the contralateral kidney									
All patients	479	284	269 (56)	157 (55)	19.8	19.9	0.98 (0.81, 1.20)	0.876	0.519
All high-risk phenotypes	367	189	228 (62)	114 (60)	22.8	23.0	0.98 (0.78, 1.23)	0.834	-
Heart failure including FPE	51	19	43 (84)	14 (73)	60.3	33.6	0.90 (0.70, 1.17)	0.446	-
Rapid progressor	67	26	46 (68)	17 (65)	25.5	25.5	0.87 (0.60, 1.26)	0.463	-
Coronary disease and diabetes	70	55	49 (70)	37 (67)	28.3	26.3	1.02 (0.64, 1.62)	0.941	-
Refractory hypertension	159	92	96 (60)	49 (53)	20.7	18.8	0.80 (0.57, 1.13)	0.215	-
eGFR <30 mL/min	213	112	141 (66)	76 (67)	26.9	26.4	0.98 (0.73, 1.30)	0.872	-
Non-high risk	112	92	41 (36)	43 (46)	11.4	14.5	1.18 (0.75, 1.83)	0.476	-
ASTRAL and Salford <70% stenosis both kidneys									
All patients	454	119	236 (51)	61 (51)	17.2	16.5	1.11 (0.83, 1.48)	0.476	0.642
All high-risk phenotypes	308	86	179 (58)	48 (55)	20.4	18.9	1.04 (0.75, 1.44)	0.817	-
Heart failure including FPE	74	13	55 (74)	8 (61)	32.6	25.2	0.75 (0.5, 1.13)	0.165	-
Rapid progressor	78	18	36 (46)	9 (50)	13.4	17.2	1.55 (0.95, 2.52)	0.077	-
Coronary disease and diabetes	99	18	57 (57)	14 (77)	20.4	27.4	1.19 (0.64, 2.24)	0.578	-
Refractory Hypertension	101	41	51 (50)	20 (48)	15.9	15.0	0.93 (0.55, 1.58)	0.795	-
eGFR <30 mL/min	162	43	106 (65)	26 (60)	25.0	24.3	1.07 (0.69, 1.66)	0.759	-
Non-high risk	146	33	57 (39)	13 (39)	11.4	11.3	1.38 (0.72,2.64)	0.33	-

Abbreviations: eGFR, estimated glomerular filtration rate; FPE, flash pulmonary edema; HR, hazard ratio.

a % per year.

A final set of analyses were undertaken in high-risk phenotypes based on CKD status. Here, people with bilateral severe disease who had either advanced CKD or who were a rapid progressor did not show benefit from revascularization with respect to likelihood of worsening of kidney function (Fig 2). In those patients who did not have bilateral severe disease, people with advanced CKD were more likely to reach a renal end point if revascularized compared with those managed medically (HR = 2.05 (1.20, 3.49), *P* = 0.009, n = 401).

## Discussion

This post hoc analysis of the ASTRAL trial, supported by data from the Salford ARVD study, suggests that people with bilateral severe RAS might benefit from kidney revascularization regardless of clinical phenotype. In both the ASTRAL analysis alone and the combined analysis with the Salford ARVD study, those with bilateral severe stenosis ≥70% had a statistically significant benefit from revascularization over medical therapy, regardless of clinical phenotype. The benefit is likely derived from cardiorenal protection and improved survival as more severe disease is associated with greater hemodynamic significance; the increased renin-angiotensin-aldosterone system stimulation and consequent inflammation, oxidative stress, and fibrosis that this brings; and thereby a higher likelihood of both de novo cardiovascular illness and worsening of existing disease.

This analysis did not reach significance for the subgroups of people with refractory hypertension or rapid progression of kidney disease in the ASTRAL and Salford combined analysis except in those with bilateral severe stenosis. However, tests for heterogeneity were nonsignificant between high-risk phenotypes. Benefit to revascularization has previously been reported in patients with both rapid progression and refractory hypertension, a combination not explored here.^14^ In a small, randomized trial of 130 patients with ARVD, revascularization was noted to confer better blood pressure control than medical therapy alone in people with bilateral stenosis.^18^ In the present analysis, we have also included a higher threshold of blood pressure in the definition of refractory hypertension compared with past studies.^10^^,^^11^ We found that revascularizing unilateral severe stenosis did not confer improved outcome even in higher risk phenotypes. This may indicate a noncausative or less predominant pathophysiological role of unilateral renal artery stenosis compared with people with bilateral disease. Kidney revascularization was not beneficial in the absence of severe bilateral RAS. Indeed, kidney revascularization for nonsevere disease was associated with worse kidney outcomes for high-risk phenotypes with advanced CKD and rapid progression. Angioplasty and stenting are known to carry procedural risks including damage to downstream organs.^10^^,^^19^ Rapid progression of CKD in the presence of only unilateral renal artery stenosis will likely represent progression of coexisting diabetic or hypertensive kidney disease.

This study has not considered the possibility that improvement after kidney revascularization might be dependent on the extent of viable renal parenchyma downstream from the stenosis.^20^ In the future, both disease severity and the assessment of kidney viability, may be better able to stratify risk and benefit from kidney intervention compared with using stenosis grade alone.^21^ Whether this stratified approach will also provide enlightenment as to the potential role of revascularization in lower risk phenotypes and lower grade or unilateral disease will only become apparent when such studies are able to be conducted. In the meantime, intervention of unilateral severe disease or bilateral moderate disease can only be advised where there is clear evidence that the stenosis is likely to be contributory to existing cardiovascular disease or unacceptable cardiovascular risk.

Another factor to be considered is the presence of albuminuria/proteinuria. In a post hoc analysis of the CORAL trial, patients were divided into groups with above and below median urine albumin-creatinine ratio at baseline (median 22.5 mg/g). In the ≤ median albuminuria group, renal artery stenting was associated with significantly better event-free survival from the primary composite end point which was similar to that used in our present analyses (73% versus 59% at 5 years; *P* = 0.02). This was not seen in the group with above median albuminuria.^22^

The limitations of the work presented are that the clinical trial analyses are post hoc and not based on prespecified subgroups. The work also includes observational data that carry greater risk of bias because those treated with revascularization may differ systematically from those not treated because of unmeasured clinical factors not included in the statistical models. Specifically, findings in the heart failure subgroup did not include ASTRAL patients so are based solely on observational data. In the combined ASTRAL and Salford analysis groups, some patients were “double counted” as 74 Salford patients were recruited to ASTRAL. Twenty percent of people randomized to revascularization in ASTRAL did not undergo the procedure. The predominantly historical nature of these cohorts means that the standard of medical management applied differs from today. Advances in optimal medical management of hypertension, CKD, and heart failure, for example the availability of SGLT2 inhibitors, means that outcomes for contemporary medically managed patients may now be better than seen in these cohorts.

To conclude, our findings indicate that the presence of bilateral severe disease may be the most important factor in patient selection for kidney revascularization. We have seen that risk of progressive loss of kidney function may actually be higher after revascularization in selected cases; thus, this as a sole intervention target may not be enough to pursue revascularization. This is also a reminder that the overall improved composite outcome even with risk of worse kidney outcomes highlights that atherosclerotic renovascular disease is primarily a systemic cardiovascular disease manifesting with poor cardiovascular outcomes. For people with bilateral severe disease, we have seen that the patient groups with the greatest apparent benefit from revascularization are those with rapidly progressing kidney dysfunction. This is in keeping with current professional guidance (including KDIGO, American Heart Association, and the European Renal Best Practice Board) that recommend consideration of the degree of stenosis in combination with the clinical phenotype.^7^^,^^8^^,^^23^

These findings appear to be important and confirmation via a randomized controlled trial is advisable. The design of such a trial should include patients with bilateral severe disease or bilateral moderate disease and high-risk phenotypes, including stage 4 CKD and heart failure.
