# Supplementary material for: Patient Selection for Revascularization of Atherosclerotic Renal Artery Stenosis: Comparing the Importance of Stenosis Severity and Clinical Phenotype
Source: Kidney Med. 2025 Dec 13;8(2):101213. doi: 10.1016/j.xkme.2025.101213 (PMC12856476; doi:10.1016/j.xkme.2025.101213)
Supplement: Supplementary File (PDF) — Tables S1-S4 [file mmc1.pdf]

Table S1. Univariate analysis of association between baseline features and composite outcome. **Bold** indicates those variables included in multivariable models of outcomes for analyses specific to each cohort.

Feature	ASTRAL		Salford		Combined	
	HR (95% CI)	p	HR (95% CI)	p	HR (95% CI)	p
Age (per year)	**1.02 (1.01,1.04)**	**<0.001**	**1.02 (1.01,1.03)**	**0.001**	**1.02 (1.01,1.03)**	**<0.001**
Female gender	0.97 (0.8,1.18)	0.78	0.94 (0.78,1.13)	0.48	0.95 (0.84,1.09)	0.488
Co-morbidities						
Coronary Disease	**1.52 (1.26,1.82)**	**<0.001**	**1.47 (1.23,1.76)**	**<0.001**	**1.49 (1.31,1.7)**	**<0.001**
Diabetes Mellitus	1.09 (0.9,1.33)	0.386	1.18 (0.98,1.43)	0.083	1.14 (0.99,1.31)	0.062
Current or ex-smoker	**1.25 (1.01,1.54)**	**0.039**	**0.77 (0.64,0.93)**	**0.007**	0.95 (0.83,1.08)	0.428
Medicines						
ACE-i or ARB	**0.82 (0.68,0.99)**	**0.039**	**0.74 (0.62,0.89)**	**0.001**	**0.78 (0.69,0.89)**	**<0.001**
Beta-blocker	**0.79 (0.66,0.95)**	**0.011**	**0.8 (0.66,0.97)**	**0.021**	**0.79 (0.69,0.9)**	**<0.001**
Anti-platelet	**1.3 (1.03,1.64)**	**0.03**	0.91 (0.76,1.09)	0.307	1.03 (0.9,1.18)	0.659
Lipid-lowering	0.96 (0.76,1.21)	0.72	**0.82 (0.68,0.98)**	**0.031**	**0.87 (0.76,0.99)**	**0.04**
Disease severity						
Systolic BP (per mmHg)	1 (1,1)	0.965	**1 (0.99,1)**	**0.012**	1 (1,1)	0.067
eGFR (per ml/min/1.73m^2)	**0.98 (0.97,0.98)**	**<0.001**	**0.97 (0.97,0.98)**	**<0.001**	**0.98 (0.97,0.98)**	**<0.001**

Table S2. Annualized event rates (percent) for each component of the composite outcome of cardiovascular and renal events or all-cause mortality in ASTRAL trial participants undergoing renal revascularization alongside standard of care, versus those only managed with medical standard of care. Findings are and displayed according to sub-groups of clinical phenotypes and stenosis severity. Key: * % per year, KRT = Kidney Replacement Therapy, CV = cardiovascular, H= hazard ratio.

Population	N		KRT*		CV event*		Death*	
	Medical	Revasc	Medical	Revasc	Medical	Revasc	Medical	Revasc
ASTRAL bilateral severe stenosis >=70%								
All patients	106	108	4.4	4.5	16.1	12.5	10.4	8.2
High risk phenotypes	83	85	5.4	5.2	19.6	13.8	10.7	8.7
Non-high risk	23	23	1.2	2.3	5.5	8.7	9.5	6.6
ASTRAL bilateral moderate or severe stenosis (≥50%)								
All patients	202	188	4.0	4.7	14.7	12.0	10.9	10.0
High risk phenotypes	161	145	5.1	5.7	16.4	14.5	11.9	11.5
Non-high risk	41	43	0.6	1.8	8.8	5.7	7.3	5.2
ASTRAL unilateral severe stenosis ≥70% with any disease <70% in contralateral kidney								
All patients	212	212	4.0	3.9	10.7	11.6	8.6	8.9
High risk phenotypes	160	138	5.1	5.2	11.5	14.6	10.5	11.3
Non-high risk	52	74	1.4	1.7	8.6	7.4	3.2	4.9

Table S3. Annualized event rates (percent) for each component of the composite outcome of cardiovascular and renal events or all-cause mortality in a combined group from the ASTRAL trial and Salford observational cohort undergoing renal revascularization alongside standard of care, versus those only managed with medical standard of care. Findings are displayed according to sub-groups of clinical phenotypes and stenosis severity. Key: * % per year, KRT = kidney replacement therapy, CV = cardiovascular, H= hazard ratio, eGFR = estimated glomerular filtration rate, FPE=flash pulmonary edema.

Population	N		KRT*		CV event*		Death*	
	Medical	Revasc	Medical	Revasc	Medical	Revasc	Medical	Revasc
ASTRAL & Salford bilateral severe stenosis >=70%								
All patients	151	152	4.8	4.3	16.7	11.5	16.3	8.3
All high-risk phenotypes	119	121	5.3	4.9	19.2	12.3	16.6	8.7
Heart failure including FPE	16	20	11.1	4.5	31.5	14.2	49.6	7.9
Rapid progressor	17	27	13.3	7.5	12.4	12.0	11.9	6.0
Coronary disease and diabetes	21	23	3.6	3.3	17.4	11.9	6.6	5.1
Refractory Hypertension	60	70	4.4	3.4	16.3	11.5	12.1	6.2
eGFR <30ml/min	70	77	7.5	7.8	26.0	11.5	19.8	11.0
Non-high risk	32	31	3.1	1.8	8.5	8.8	15.1	6.8
ASTRAL & Salford bilateral moderate or severe stenosis >=50%								
All patients	381	277	4.2	4.2	13.2	11.6	13.4	9.7
All high-risk phenotypes	298	212	5.1	5.2	14.7	13.3	14.8	11.1
Heart failure including FPE	39	32	8.0	4.1	28.8	16.2	32.6	11.4
Rapid progressor	54	41	8.7	7.3	12.6	15.1	11.3	10.1
Coronary disease and diabetes	65	45	5.6	4.2	14.7	14.1	15.2	6.4
Refractory Hypertension	145	113	4.2	3.8	13.6	12.6	10.9	8.3
eGFR <30ml/min	182	123	6.8	8.9	17.1	12.5	17.7	15.1
Non-high risk	83	62	1.7	1.3	8.6	7.3	9.2	5.9
ASTRAL & Salford unilateral severe stenosis >=70% with any disease <70% in the contralateral kidney								
All patients	479	284	4.2	3.7	9.8	11.4	11.5	9.5
All high-risk phenotypes	367	189	5.2	4.7	11.2	12.8	13.2	11.7
Heart failure including FPE	51	19	12.2	4.0	21.3	11.0	31.7	18.6
Rapid progressor	67	26	7.2	10.2	16.2	15.4	11.3	13.0
Coronary disease and diabetes	70	55	4.4	3.1	15.9	16.5	16.9	11.5
Refractory Hypertension	159	92	4.9	3.4	11.3	11.7	10.4	8.0
eGFR <30ml/min	213	112	8.2	8.2	10.9	12.3	16.6	14.7
Non-high risk	112	92	1.4	2.0	5.9	9.1	6.7	5.8
ASTRAL & Salford <70% stenosis both kidneys								
All patients	454	119	3.1	3.2	8.9	8.4	11.0	8.3
All high-risk phenotypes	308	86	4.2	4.3	10.3	8.9	12.9	9.8

Heart failure including FPE	74	13	4.0	5.0	17.1	20.4	18.6	15.0
Rapid progressor	78	18	3.0	4.8	8.2	10.0	6.9	9.9
Coronary disease and diabetes	99	18	4.1	6.6	11.0	10.8	13.9	12.8
Refractory Hypertension	101	41	4.1	3.1	10.0	9.2	10.1	7.0
eGFR <30ml/min	162	43	6.3	8.9	11.3	6.7	15.6	15.7
Non-high risk	146	33	1.1	0.8	6.2	7.0	7.3	4.5

Table S4. Hazard ratios for the composite outcome of cardiovascular and renal events or all-cause mortality in a combined group from the ASTRAL trial and Salford observational cohort undergoing renal revascularization alongside standard of care, versus those only managed with medical standard of care. Findings are adjusted for baseline age, eGFR, coronary disease, smoking, and use of RAASi, betablockers and antiplatelet therapy, and displayed according to sub-groups of clinical phenotypes and stenosis severity. Key: * % per year, CV = cardiovascular, HR = hazard ratio, eGFR = estimated glomerular filtration rate, FPE=flash pulmonary edema, na = heart failure data not available.

Population	Events (n, %)		Event rate*		HR [95% CI]	p
	Medical	Revasc	Medical	Revasc		
ASTRAL bilateral severe stenosis >=70%						
All patients	70 (66)	63 (58)	25.9	19.9	0.70 (0.50,1.00)	0.048
All high-risk phenotypes	59 (71)	52 (61)	29.8	22.1	0.70 (0.48,1.03)	0.070
Heart failure including FPE	na	na	na	na	na	na
Rapid progressor	8 (66)	13 (65)	33.6	20.7	0.12 (0.03,0.54)	0.005
Coronary disease and diabetes	12 (63)	10 (62)	21.4	19.8	1.21 (0.47,3.06)	0.694
Refractory Hypertension	37 (72)	29 (53)	28.2	18.4	0.60 (0.35,1.00)	0.051
eGFR <30ml/min	40 (76)	35 (68)	39.3	26.8	0.60 (0.36,0.99)	0.044
Non-high risk	11 (47)	11 (47)	15.2	13.6	0.50 (0.20,1.28)	0.150
ASTRAL bilateral moderate or severe stenosis >=50%						
All patients	129 (63)	112 (59)	24.6	21.0	0.85 (0.66,1.10)	0.218
All high-risk phenotypes	108 (67)	96 (66)	27.1	25.3	0.91 (0.69,1.21)	0.522
Heart failure including FPE	na	na	na	na	na	na
Rapid progressor	22 (75)	20 (68)	35.7	25.8	0.49 (0.24,0.99)	0.047
Coronary disease and diabetes	25 (62)	19 (63)	21.8	22.2	0.92 (0.48,1.75)	0.801
Refractory Hypertension	63 (70)	49 (58)	27.8	20.1	0.72 (0.49,1.05)	0.090
eGFR <30ml/min	71 (72)	65 (74)	34.4	32.4	1.01 (0.72,1.42)	0.940
Non-high risk	21 (51)	16 (37)	16.9	10.4	0.58 (0.30,1.15)	0.121
ASTRAL unilateral severe stenosis >=70% with any disease <70% in the contralateral kidney						
All patients	118 (55)	117 (55)	18.2	19.5	1.06 (0.82,1.37)	0.663
All high-risk phenotypes	100 (62)	85 (61)	21.1	24.0	1.09 (0.81,1.46)	0.568
Heart failure including FPE	na	na	na	na	na	na
Rapid progressor	26 (83)	13 (68)	35.7	30.2	0.65 (0.32,1.34)	0.246
Coronary disease and diabetes	23 (62)	31 (68)	23.2	28.7	1.18 (0.66,2.11)	0.570
Refractory Hypertension	48 (64)	38 (53)	22.2	18.2	0.73 (0.47,1.13)	0.157
eGFR <30ml/min	64 (64)	58 (66)	23.7	27.1	1.18 (0.80,1.73)	0.410
Non-high risk	18 (34)	32 (43)	10.3	13.1	1.27 (0.70,2.03)	0.440
ASTRAL & Salford <70% stenosis both kidneys						

All patients	43 (53)	41 (50)	17.3	15.7	0.97 (0.62,1.52)	0.899
All high-risk phenotypes	30 (61)	28 (52)	21.8	16.9	0.81 (0.47,1.40)	0.458
Heart failure including FPE	na	na	na	na	na	na
Rapid progressor	2 (33)	5 (55)	10.0	20.1	0.94 (0.04,19.7)	0.968
Coronary disease and diabetes	9 (56)	6 (66)	17.5	22.2	1.26 (0.33,4.75)	0.733
Refractory Hypertension	16 (66)	12 (42)	26.3	12.6	0.54 (0.24,1.22)	0.139
eGFR <30ml/min	20 (71)	16 (59)	27.4	20.6	0.86 (0.42,1.73)	0.667
Non-high risk	13 (40)	13 (44)	11.8	13.7	1.76 (0.74,4.21)	0.204

Table S5. Hazard ratios for the composite outcome of cardiovascular and renal events or all-cause mortality in the Salford observational cohort undergoing renal revascularization alongside standard of care, versus those only managed with medical standard of care. Findings are adjusted for baseline age, eGFR, coronary disease, smoking, systolic blood pressure, use of RAASi, beta-blockers and lipid lowering therapy, and displayed according to sub-groups of clinical phenotypes and stenosis severity. Key: * % per year, HR= hazard ratio, eGFR = estimated glomerular filtration rate, FPE=flash pulmonary edema.

Population	Events (n, %)		Event rate*		HR [95% CI]	p
	Medical	Revasc	Medical	Revasc		
Salford bilateral severe stenosis >=70%						
All patients	38 (84)	23 (52)	60.6	19.4	0.44 (0.23,0.86)	0.017
All high-risk phenotypes	30 (83)	21 (58)	54.7	21.4	0.48 (0.24,0.99)	0.045
Heart failure including FPE	15 (93)	13 (65)	85.8	27.2	0.13 (0.03,0.63)	0.011
Rapid progressor	3 (60)	4 (57)	18.8	17.5	0.43 (0.19,0.99)	0.049
Coronary disease and diabetes	2 (10)	3 (42)	43.7	14.4	0.31 (0.01,6.51)	0.447
Refractory Hypertension	7 (77)	8 (50)	31.4	16.5	0.40 (0.11,1.44)	0.162
eGFR <30ml/min	22 (88)	10 (52)	78.7	17.3	0.26 (0.11,0.57)	0.001
Non-high risk	8 (88)	2 (25)	101.4	9.8	0.08 (0.01,1.06)	0.055
Salford bilateral moderate or severe stenosis >=50%						
All patients	106 (64)	45 (52)	26.5	19.5	0.75 (0.51,1.10)	0.136
All high-risk phenotypes	87 (67)	38 (58)	29.2	22.4	0.77 (0.51,1.17)	0.224
Heart failure including FPE	35 (94)	20 (64)	75.0	29.8	0.39 (0.19,0.79)	0.010
Rapid progressor	12 (48)	7 (58)	13.0	21.3	0.84 (0.52,1.35)	0.472
Coronary disease and diabetes	18 (72)	9 (60)	33.0	22.0	0.73 (0.27,1.98)	0.541
Refractory Hypertension	26 (52)	15 (53)	17.1	18.4	1.18 (0.59,2.33)	0.640
eGFR <30ml/min	57 (70)	19 (55)	33.7	20.0	0.61 (0.36,1.04)	0.071
Non-high risk	19 (52)	7 (33)	18.5	11.4	2.16 (0.69,6.78)	0.188
Salford unilateral severe stenosis >=70% with any disease <70% in the contralateral kidney						
All patients	151 (58)	40 (56)	21.4	21.1	1.01 (0.7,1.45)	0.954
All high-risk phenotypes	128 (63)	29 (56)	24.5	20.8	0.88 (0.58,1.32)	0.534
Heart failure including FPE	43 (86)	14 (73)	60.3	33.6	0.43 (0.20,0.93)	0.033
Rapid progressor	20 (52)	4 (57)	18.6	16.9	1.03 (0.68,1.54)	0.902
Coronary disease and diabetes	26 (76)	6 (60)	35.1	18.5	0.51 (0.19,1.36)	0.176
Refractory Hypertension	48 (58)	11 (52)	19.5	21.4	1.37 (0.72,2.61)	0.337
eGFR <30ml/min	77 (67)	18 (69)	30.4	24.5	0.78 (0.46,1.32)	0.356
Non-high risk	23 (39)	11 (55)	12.5	21.8	1.80 (0.71,4.58)	0.217
Salford <70% stenosis both kidneys						
All patients	151 (41)	40 (11)	21.4	21.1	1.15 (0.72,1.86)	0.552
All high-risk phenotypes	128 (50)	29 (93)	24.5	20.8	1.28 (0.79,2.08)	0.319

Heart failure including FPE	43 (59)	14 (11)	60.3	33.6	0.89 (0.40,1.96)	0.765
Rapid progressor	20 (28)	4 (44)	18.6	16.9	1.37 (0.80,2.34)	0.247
Coronary disease and diabetes	26 (31)	6 (66)	35.1	18.5	1.08 (0.43,2.70)	0.873
Refractory Hypertension	48 (64)	11 (91)	19.5	21.4	1.42 (0.64,3.13)	0.384
eGFR <30ml/min	77 (58)	18 (12)	30.4	24.5	1.05 (0.52,2.13)	0.886
Non-high risk	23 (20)	11 (27)	12.5	21.8	0 (0,2.77)	0.957
